# Characterization of microbial communities in seven wetlands with different anthropogenic burden using Next Generation Sequencing in Bogotá, Colombia

**DOI:** 10.1038/s41598-023-42970-w

**Published:** 2023-10-09

**Authors:** Nathalia Ballesteros, Luisa Páez, Nicolas Luna, Ariana Reina, Vanessa Urrea, Catalina Sánchez, Angie Ramírez, Juan David Ramirez, Marina Muñoz

**Affiliations:** 1 Centro de Investigaciones en Microbiología y Biotecnología-UR (CIMBIUR), Facultad de Ciencias Naturales, Universidad del Rosario, Bogotá, Colombia; 2 Departamento de Biología, Facultad de Ciencias Naturales, Universidad del Rosario, Bogotá, Colombia; 3 Molecular Microbiology Laboratory, Department of Pathology, Molecular and Cell-Based Medicine, Icahn School of Medicine at Mount Sinai, New York City, NY USA

**Keywords:** Microbial ecology, Microbiology, Microbial communities

## Abstract

Wetlands represent key ecosystems due to their remarkable biodiversity, ecological functions and multiple ecosystem services provided. In Colombia, there are 31,702 wetlands, 13 of which are in Bogotá, capital of the country. Despite the fundamental socioecological support of these aquatic ecosystems, a tremendous loss and degradation of these ecosystems has been observed due to anthropogenic perturbations. Therefore, the aim of this study was to describe the status of seven Bogotá wetlands with variable anthropogenic interventions by measuring organoleptic, physicochemical, and microbiological parameters, using commercial kits, highly sensitive equipment, and next-generation sequencing of the 16S- and 18S-rRNA genes. Our findings describe the status of seven wetlands with different anthropogenic burden in Bogotá-Colombia where physicochemical and microbiology signals of contamination were observed. Additionally, some profiles in the composition of the microbial communities, together with certain physicochemical characteristics, may represent an insight into the environmental dynamics, where Beta Proteobacteria such as *Malikia* represent a potential keystone in aquatic ecosystems impacted by wastewater effluent discharges; the presence of nitrates and phosphates explain the abundance of bacteria capable of oxidizing these compounds, such as *Polynucleobacter*. Moreover, the presence of specific prokaryotic and eukaryotic organisms, such as *Clostridium*, *Cryptococcus*, *Candida*, and *Naegleria*, reported in one or more of the wetlands assessed here, could represent a possible pathogenic risk for human and animal health. This study performed a complete evaluation of seven Bogotá wetlands with different anthropogenic impacts for the first time, and our findings emphasize the importance of maintaining continuous monitoring of these water bodies given their remarkable ecological importance and potential spill-over of several pathogens to humans and animals.

## Introduction

Wetlands comprise a broad array of water bodies with geo-morphological and hydrological conditions that allow water accumulation and include natural or artificial, permanent or temporary, stagnant or flowing, fresh, brackish, or salty bodies of water^1^. They are ecosystems of significant natural and cultural value since they provide essential water for the maintenance of flora and fauna^2^. Additionally, wetlands are essential for regulating the water cycle, purifying the air, providing habitat for endemic species and migratory birds, and acting as carbon sinks^3^. In Colombia, there are 31,702 reported wetlands, with 13 of these located in Bogotá, the country’s capital and most populous city^4^. However, due to their location, some of these wetlands have experienced environmental disturbances in recent years due to increased urbanization and pollution generated by the surrounding communities^5^. This implies that anthropogenic activities such as wastewater dumping, waste material falling into water sources, urban construction, and other alterations and transformations of the ecosystem are significant factors contributing to the loss of water quality of these aquatic ecosystems^6,7^. Thus, quality refers to the measure of the chemical, physical, and biological characteristics of water that meet the conditions required by one or more biotic species for a specific use^8^.

Water quality assessment has been highly implemented worldwide to determine the status of water resources for human consumption. Therefore, organoleptic, physicochemical, and biological parameters are fundamental in characterizing water sources. The measurement of organoleptic variables such as color and odor, and physicochemical variables such as temperature, pH, presence of organic matter, alkalinity, etc., are adjusted to quality indexes and allow constant monitoring of water resources to take actions and mitigate sanitary risk^8^. Regarding biological variables, the search for bioindicators and pathogenic microorganisms in terms of public and environmental health has been partially explored in water bodies^9^. Some microorganisms of special interest due to their pathogenic potential are bacteria such as *Escherichia coli* and other coliforms^10^, some protozoa such as *Giardia*, *Cryptosporidium*^10^, *Cyclospora*^11^ and *Acanthamoeba*^12^, and helminths like *Schistosoma* spp.^13^. Thus, bacterial and eukaryotic communities represent useful tools to understand the richness that encompasses these ecosystems, considered as one of the factors that condition the fertility, stability and functioning of the different environments^14^.

The implementation of Next Generation Sequencing (NGS), specifically the use of amplicon-based sequencing of hypervariable regions of bacterial (16S-rRNA) and eukaryotic (18S-rRNA) communities, constitutes a first-hand tool for characterizing the microbial composition of an ecosystem^15^. Some studies have employed this approach in water bodies with varying levels of anthropogenic burden to describe changes in the community structure of bacteria, archaea and eukaryotes^16^. These studies have identified bacteria belonging to the phyla Bacteroidetes, Acidobacteria, and Firmicutes^7,16^, as well as pathogenic microorganisms such as *Pseudomonas alcaligenes, Clostridium perfringens*^7^, *Giardia duodenalis*^17^, *Cryptosporidium,* and *Aspergillus*, among others^7^. In Colombia, a study of urban recreational lakes and a stream in Bogotá, using high-throughput 16S- and 18S-rRNA gene sequencing along with assessment of physicochemical characteristics, reported the possible public health threat posed by these ecosystems due to the presence of some bacterial pathogens including *Legionella*, *Pseudomonas* and *Mycobacterium*, and eukaryotic pathogens such as *Candida* and *Naegleria*^18^. This highlights the potential application of this methodology for high-resolution monitoring of water quality and the state of water bodies related to environmental and/or anthropogenic burden.

Given the essential services provided by wetlands, an intergovernmental treaty denominated “Ramsar Convention on Wetlands” was adopted to conserve and sustainably utilize wetlands and their resources^19^. In response to the convention, a List of Wetlands of International Importance was created, which currently includes over 2400 wetlands recognized internationally for their significant value^19^. In Colombia, as of 2022, nine wetlands (760,340 hectares) have been designated as Ramsar sites, one of which is the Complex of Urban Wetlands located in Bogotá^19^. Despite the growing national and international concern for the loss and degradation of these ecosystems due to external factors such as industrialization, urbanization, changes in the land use, there is a lack of knowledge in Colombia regarding the ecological state of these ecosystems, the composition and abundance of microbial communities that could act as key indicators of the ecosystem health, adequate ecological function, the quality of the water, and moreover, the possible presence of pathogenic microorganisms that could threaten human and animal health. Therefore, the present study aims to compare the status of seven wetlands with different levels of anthropogenic burden by measuring organoleptic, physicochemical, and microbiological parameters with the objective of providing an unprecedented biological and ecological description of these water bodies with varying human burden.

## Methods

### Sample collection and physicochemical analysis

**Figure 1:**
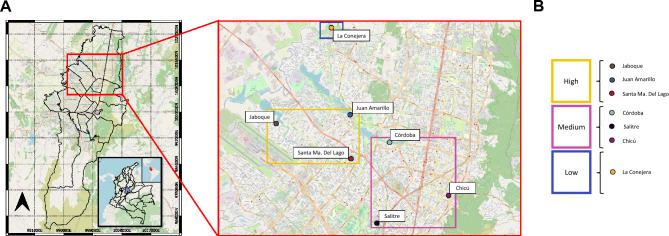
Geographic location of sampling sites included in the study. (**A**). From left to right: map of Colombia, Cundinamarca, and Bogotá D.C. (**B**). Wetlands classification by level of anthropogenic intervention.

Seven wetlands located in urban areas of the city of Bogotá, central Colombia, were selected: Chicú (4.674917, − 74.044066), covering an area of 0.025 ha; La Conejera (4.7616667, − 74.1049999) covering an area of 58.9 ha; Córdoba (4.702517, − 74.074748) covering an area of 40.5 ha; Juan Amarillo (4.717, − 74.095) covering an area of 222.76 ha; Salitre (4.660698, -74.081086) covering an area of 3.42 ha, Santa Ma. del Lago (4.694, − 74.095) covering an area of 5.65 ha; and Jaboque (4.7122222, − 74.133611) covering an area of 148 ha (Fig. 1). An initial description of the sampling site was carried out, considering variables such as altitude, ambient temperature, and the general state of the area, including landuse, surrounding vegetation, appearance of the source, geographic coordinates, proximity to urban settlements, and other anthropogenic burden (Supplementary Information 1).

The water samples were collected in November 2020 after notifying the corresponding environmental authority and following existing environmental regulations (Supplementary Information 2). During this month, the Instituto de Hidrología, Meteorología y Estudios Ambientales (IDEAM) reported an accumulation of precipitation for Bogotá D.C., in the range between 80 and 100 mm. Organoleptic properties of the water were registered in situ, such as color, smell, and the presence of chemicals and decomposing organic matter. Composite water samples were collected at a depth of 20 cm in sterile plastic containers with a capacity of 500 mL, by collection of the water at three different points of the water body to obtain the most accurate representation of the biotic and abiotic characteristics. Physicochemical parameters were evaluated according to the national resolution 2115 of 2007^10^. To do so, alkalinity, acidity, and hardness were determined in situ using the “Carolina® Water Quality Saddlebag” kit. In laboratory, pH, percentage of dissolved oxygen, and carbon dioxide (ppm) were measured using the LabQuest2 multiparameter equipment.

Additionally, a traditional microbiological analysis was performed. The presence of total coliforms was verified through serial dilutions up to 1 × 10^–3^ in sterile distilled water. After this, massive seeding was carried out on nutrient agar and MacConkey agar plates with the odd dilutions (1 × 10^–1^ and 1 × 10^–3^), and subsequently incubated at 37 °C for 48 h to assess the presence of a broad range of bacteria and more selectively assess the presence of Gram-negative bacteria. Afterwards, colony-forming units (CFU) were counted to determine compliance with the maximum acceptable values described in the national resolution 2115^10^.

### Anthropogenic burden determination

To determine the level of anthropogenic intervention, we used the specific coordinates of the sample collection points in the Environmental Geographic Viewer^20^, tool designed by the Secretaría Distrital de Ambiente of Bogotá, for consolidating and analyzing environmental data. This environmental authority carried out periodic monitoring of air, soil and water quality variables, vegetation, climate change related variables, and population density parameters, which were analyzed and summarized in a table (Supplementary Information 3). Subsequently, a score was assigned per variable. For example, the wetland with the highest value for Mean PM10 was assigned a highest value, followed by the assignment of values to the other wetlands considering their respective Mean PM10. This assignment was performed for all the variables analyzed and include in the Supplementary Information 3. Finally, a final score was calculated by summing up the values of each variable, and the wetlands were classified based on their level of anthropogenic burden as high, medium, or low.

### DNA extraction and axmplicon-based sequencing of the V4 hypervariable region of the 16S-rRNA and 18S-rRNA genes

The water samples were filtered using Cellulose Acetate (CA) Membrane Filters with different pore size, including 3 µm, 0.45 µm and 0.20 µm, to capture all different-sized biological material present in the sample. Afterwards, the biomass was mechanically recovered from all the filters used per sample with Nuclease Free Water to finally performed the DNA extraction from the pellet obtained. The total DNA was obtained following the manufacturer’s instructions of the commercial kit DNeasy PowerSoil Kit from Qiagen.

For each sample, the V4 hypervariable region of the 16S-rRNA and 18S-rRNA genes were amplified using the primers 515F 5’-GTGCCAGCMGCCGCGGTAA-3’and 806R 5’-GGACTACHVGGGTWTCTAAT-3’^21^, and 528F 5’-GCGGTAATTCCAGCTCCAA-3’ and 706R 5’-AATCCRAGAATTTCACCTCT-3’^22^, respectively. The amplification of each molecular marker was carried out under the PCR conditions previously reported^23^. Amplicon-based sequencing was performed using the Illumina Novaseq 6000 platform with paired-end reads of 250 bp length (Supplementary Information 4), the best DNA quality criteria and an expected raw read depth of 100,000 reads.

### Quality control of reads and obtaining of ASVs

After sequencing, we obtained raw paired-end de-multiplexed sequences without primers and adapters. We checked the quality of these reads through a FastQC version 0.11.7^24^ and consolidated and visualized the data with MultiQC version 1.6^25^. We performed taxonomic assignment of the amplicon sequence variant (ASVs) using version 1.16 of the DADA2 (Divisive Amplicon Denoising Algorithm) package (Callahan et al., 2016) in R software version 4.0.2 (R Core Team, 2022). For this package, we used the recommended parameters of the microbiome analysis pipeline (https://benjjneb.github.io/dada2/tutorial.html). The pipeline filters individual reads considering a Phred score equal to or higher than 30 to minimize misreads, merges forward and reverse sequences, and infers the amplicon sequence variants (ASVs), which are defined as different unique sequences^26^. After obtaining the ASVs, we removed the chimeric structures of the sequences. Finally, with DADA2, we taxonomically assigned each ASV by comparing it with SILVA version 138^27^ and PR2 version 4.14.0^28^ databases for 16S- and 18S-rRNA markers, respectively.

### Wetland microbiota composition and diversity

From the abundance and taxonomic assignment tables, we filtered out the ASVs corresponding to mitochondria, chloroplast, and eukaryote for 16S-rRNA and metazoa and algae for 18S-rRNA. To visually inspect the total richness of our samples, we generated rarefaction curves using phyloseq^29^ and ampvis2^30^ R packages. Furthermore, we rarified our data to equal sampling depth using the function *rarefy_even_depth* (rngseed = 1) from *phyloseq* to normalize differences in sampling depths between samples that can influence dissimilarity metrics^40^.

To describe microbial communities, we identified the ten most abundant genera using phyloseq^29^ and microbiome^31^ packages based on the proportion of reads of each ASV to the total (relative abundance) of the dataset for each wetland. The diversity of ASVs for each wetland habitat (alpha diversity) was calculated using the Shannon–Wiener (species diversity) and Chao1 (richness) indices of the R microbiome package^31^. Finally, to evaluate the dissimilarity between wetlands (beta diversity) and how environmental variables are associated with the microbial composition, a constrained analysis of principal co-ordinates (CAP) was conducted with the R package Vegan^32^.

### Identification of pathogens

We performed additional analyses for taxa of interest due to their importance in the ecosystem and public health^33^. For this purpose, we performed a BLASTn to improve the precision of the taxonomic assignation to species level. For this process, we generated a reference database for each genus/phylum, with the available sequences of the V4 hypervariable region of the 16S and 18S genes in the curated database RefSeq^34^, and for the specific case of *Blastocystis*, we constructed a database was made with the 18S-rRNA sequences available in PubMLST. For the taxonomic assignment through BLASTn, we considered a minimum 90% identity and e-value of 10 as a match. The best 5 matches were obtained, and the best result was selected. The abundances of these taxa by wetland were represented in a heatmap with the R package Phyloseq^29^.

## Results

### Physicochemical characteristics of the water bodies

The physicochemical characteristics of seven wetlands located in the urban area of Bogotá D.C., Colombia, were evaluate. The geographical location of each wetland is shown in Fig. 1A, demonstrating the proximity to urban settlements. However, the effect of wetland management type was evident, with those with restricted public access presenting better conditions (i.e., Santa Ma. del Lago and Chicú) and less anthropogenic impact. Conversely, wetlands highly surrounded by human settlements with open access presented greater contamination by solids, with waste discharge from nearby communities and a direct impact by informal settlements of migrants within the wetland who make direct use of the water body of the ecosystem (i.e., Juan Amarillo). The organoleptic properties observed for all the wetlands were the apparent color, turbidity, the smell of earth and leaves, the absence of observable chemicals, and the presence of decomposing organic matter.

On the other hand, the Secretaría Distrital de Ambiente analyzed the environmental variables giving us a greater insight into direct and indirect anthropogenic burden. The analysis of these variables enables the classification of seven wetlands with high, medium, and low human pressured (Fig. 1B and Supplementary Information 3). Wetlands classified with a high anthropogenic burden (Jaboque, Juan Amarillo and Santa Ma. Del Lago) presented higher values of PM10, especially, the Jaboque wetland (Supplementary Information 3). Furthermore, these high-impacted ecosystems are characterized by a higher population density, with more inhabitants per Km^2^ and low soil quality related to proximity to industrial, hospital and construction waste generators and sites of final disposition of construction and demolition waste. On the contrary, the low-pressured wetland, La Conejera, is characterized by a complex vegetation cover (forest) and a high quantity of trees per hectare (Supplementary Information 3).

The physicochemical characteristics are summarized in the Table 1. The pH of all the wetlands ranged between 6.5 and 7.38, with the lowest value in La Conejera wetland and the highest value for Juan Amarillo and Salitre wetlands. Additionally, La Conejera wetland showed the lowest value for the dissolved O_2_ with 3.64 ppm, the highest value of the dissolved CO_2_ (751 ppm), and one of the few wetlands assessed with presence of nitrites and nitrates. Conversely, the Chicú and the Salitre wetlands presented the highest value for dissolved O_2_ and the lowest value for dissolved CO_2_, respectively. Moreover, the Salitre wetland presented the highest value for hardness and nitrites parameters, the Jaboque wetland presented the highest value for the alkalinity and the lowest value for the hardness, with a reported value of zero (Table 1). Finally, only the nitrite values for La Conejera, Salitre and Jaboque wetlands were above the maximum acceptable values described in the national resolution 2115, and therefore with possible implications for human health. However, there is not a clear association of the physicochemical variables with the levels of anthropogenic burden and neither a statistical difference between them.

**Table 1:** Measured physicochemical characteristics in the seven water bodies.

Anthropogenic interference	Wetland	Environmental temperature (ºC)	pH	CaCO3 (ppm*)			Dissolved O2 (ppm*)	Dissolved CO_2_ (ppm*)	Nitrites	Nitrates
				Alcalinity	Acidity	Hardness				
High	Jaboque	19	6.66	120	30	0	4	678	4	1
	Juan Amarillo	22	7.38	40	4	30	12.46	433	0	0
	Santa Ma. del Lago	22	6.79	60	10	60	11.55	543	0	0
	Mean	21	6.9	73.3	14.7	30	9.3	551.3	1.3	0.3
Medium	Córdoba	15	6.88	60	26	40	12.94	523	0	0
	Salitre	17	7.38	110	40	80	12.81	432	5	0.5
	Chicú	16	6.57	20	20	20	13.04	493	0	0
	Mean	16	6.9	63.3	28.7	47	12.9	482.7	1.7	0.2
Low	La Conejera	17	6.5	50	24	50	3.64	751	2	0.3

*ppm: parts per million.

In the traditional microbiological analysis, bacterial growth was observed in both types of agars used (Nutrient Agar and MacConkey Agar). The values of the counts obtained were different for each wetland. Counts were obtained from 0 CFU/mL to highly numerous to count (> 6500), results are registered in Table 2. In general, bacterial growth in nutrient agar were colonies of different sizes, with white or yellow colors, aerobes that correspond to coliforms, possibly *Escherichia coli*. Additionally, in Salitre and Jaboque wetlands showed pink colonies in MacConkey agar. These colonies correspond to lactose-fermenting bacteria, also representing metabolic characteristics of *E. coli*. The wetlands with higher CFU and with a higher presence of coliforms/*E. coli* colonies were Córdoba and Jaboque. In contrast, the wetlands with fewer CFU observed were Santa Ma. del Lago and Salitre, representing better water quality. On average, the highly and medium pressured wetlands present more CFU in both agars than the low pressured wetland, highlighting a possible relationship between the human impact in the ecosystem and a major presence of coliforms/*E. coli*.

**Table 2:** Traditional microbiological analysis. Count of colony forming units (CFU).

Anthropogenic interference	Wetland	Nutrient agar (CFU/mL)	MacConkey agar (CFU/mL)
High	Jaboque	810	910
	Juan Amarillo	520	280
	Santa Ma. del Lago	30	0
	Mean	453.33	396.67
Medium	Córdoba	> 6500	> 6500
	Salitre	110	30
	Chicú	170	60
	Mean	2260	2196.67
Low	La Conejera	120	170

### Description of the prokaryotic and eukaryotic communities

Regarding the diversity of the microbial communities using Amplicon Sequence Variants (ASV), it varied between wetlands and markers (16S and 18S) (Fig. S1). The observed bacteria ASV range from approximately 270 to over 500 ASVs, with Jaboque showing the smallest number of ASV, and Juan Amarillo showing the most abundant ASV. Conversely, the observed eukaryotic ASV range from 70 to 370, corresponding to Jaboque and Chicú wetlands, respectively.

**Figure 2:**
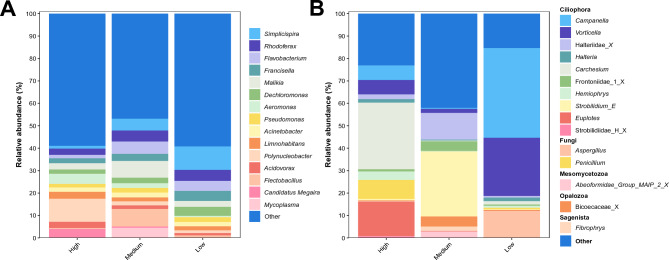
Relative abundance of microbial genera in each wetland categorized by anthropogenic burden (low, medium, high) for (**A**). the prokaryotic community (based on 16S-rRNA amplicon sequencing) and (**B**). the eucaryotic community (based on 18S-rRNA amplicon sequencing.

Taxonomic identification of bacterial communities showed a similar panorama with the presence of some bacterial genera in the three types of wetlands (Fig. 2A), but with different relative abundances. La Conejera showed a major relative abundance in the genera *Simplicispira*, *Francisella*, and *Dechloromonas*; the medium-pressured wetlands had a major abundance in the genera *Flavobacterium*, *Malikia* and *Flectobacillus*, additionally, these were characterized by the presence of the genus *Mycoplasma.* Finally, the high-pressured wetlands had an increased abundance of the genera *Polynucleobacter*, *Acidovorax*, and, especially, *Candidatus megaira* (Fig. 2A). The organisms belonging to the phylum Cyanobacteria were highly abundant, mainly in Cordoba and Jaboque wetlands (Fig. S2). Due to limitations in publicly available sequences, taxonomic assignment to the family level or higher precision was not possible for this group of data. Consequently, we excluded this group from the abundance analysis to enhance the visualization of the remaining organisms.

In terms of the eukaryotic diversity, the phyla Ciliophora, Fungi, Mesomycetozoa, Opalozoa and Sagenista were mostly abundant (Fig. 2B). Specifically, each type of wetland had a different eukaryotic genera composition, with some more predominant than others; in the case of low anthropogenic burden wetlands, a great proportion of reads were classified as *Campanella*, *Vorticella*, and *Aspergillus*. The medium-pressured wetlands were characterized by an abundance increase in the genera *Halteridae*, *Frontoniidae*, *Strobilidium*, and *Bicoecaceae*. Lastly, the highly intervened wetlands were characterized by the presence of *Carchesium*, *Euplotes* and *Penicillium* (Fig. 2B). The majority of these microorganisms represent free-living ciliates that are part of the microbiota of aquatic ecosystems^35^, with the others representing free living fungi.

**Figure 3:**
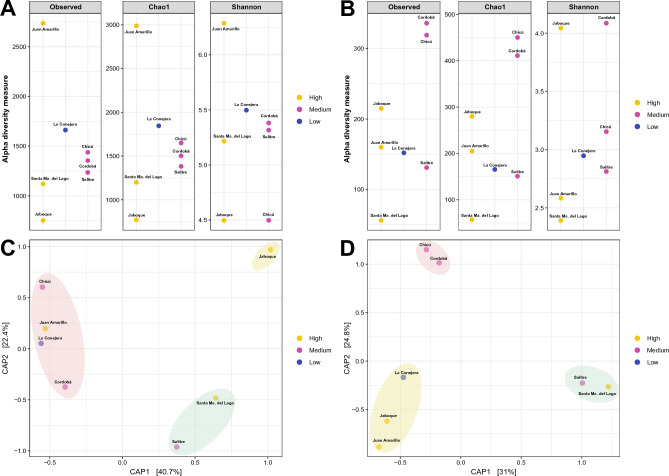
Alpha Diversity of the different wetlands. (**A**). For the procaryotic community (16S-rRNA). (**B**). For the eucaryotic community (18S-rRNA). Beta diversity of the wetland and their respective aggrupation. (**C**). For the procaryotic community (16S-rRNA). (**D**). For the eucaryotic community (18S-rRNA).

Additionally, alpha diversity was assessed, Fig. 3, representing the diversity and dominance of the prokaryotic and eukaryotic communities (Fig. 3A and B) using the Shannon and Simpson indexes, respectively. For both microbial communities, various taxonomic groups were presented, represented by the Shannon value (microbial diversity), with a high microbial richness (Chao1 value) which support the diversity observed. Beta diversity showed three groups with different composition between the 16S- and 18S-rRNA markers (Fig. 3C and D). In the prokaryotic composition, the Chicú, Juan Amarillo, La Conejera and Cordoba wetlands were similar and represented one of the groups. The second group was composed of the Santa Ma. del Lago and the Salitre wetland, while the Jaboque wetland represented the last group (Fig. 3C). In the beta diversity analysis of the eukaryotic composition, the largest group was composed by the Santa Ma. del Lago, La Conejera, Cordoba and Salitre wetlands, the second group by the Juan Amarillo and Chicú wetlands, and similarly to the prokaryotic community, the Jaboque wetland represents a different group (Fig. 3D). However, neither the alpha diversity nor the beta diversity analysis showed a similar behavior within the differentially anthropogenic pressured wetlands.

**Figure 4:**
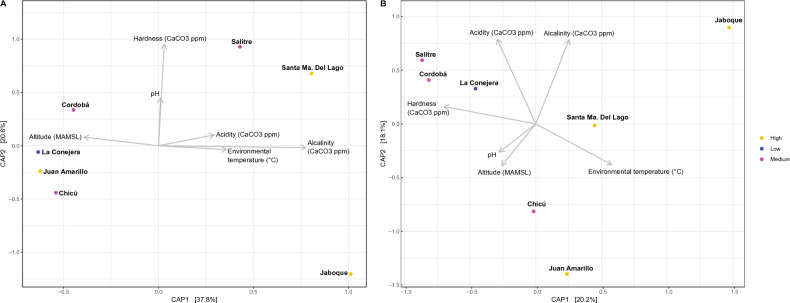
Beta diversity of each group of wetlands and the environmental variables associated with the changes in community composition. (**A**). For the procaryotic community (16S-rRNA). (**B**). For the eucaryotic community (18S-rRNA).

Nevertheless, the beta diversity clusters were evaluated with the physicochemical variables to determine the possible associations of these external factors and changes in the microbial community composition (Fig. 4). Regarding eukaryotes, our analysis gives an insight of possible associations of microbial composition with the physicochemical factors, principally of the first group with hardness, acidity, and alkalinity, the second group with altitude, pH, and temperature, and the Jaboque wetland presented a combination of different physicochemical characteristics that could explain its discriminatory microbial composition (Fig. 4B). Similarly, in prokaryotes, the first group could be associated with altitude (mean 2591.5 m above sea level, SD = 40.8), the second group with pH and hardness variables, consisting of the highest hardness values registered (Table 1), and the Jaboque wetland (third group) that could be associated principally with the alkalinity (Fig. 4A), possessing the highest value registered out of all the wetlands (Table 1).

Furthermore, potential prokaryotic and eukaryotic pathogenic microorganisms were assessed (Fig. S3). For prokaryotes, there was a presence of *Clostridium *sensu* strictu* and *Lepstospira* in low abundance, however, there was a direct correlation between the level of anthropogenic burdenand the abundance of *Aeromonas* and *Escherichia-Shigella* (Fig. S3A), with highly pressured wetlands showing a higher proportion of these pathogens than the low-pressured ones. For eukaryotes, there was a minor diversity and abundance of pathogenic microorganisms, with *Blastocystis* abundance (specifically the subtype 17 with an e-value of 6.14e−19) being shared across all types of wetlands. However, there were different percentages of abundance for *Candida* (*Candida intermedia* with an identity percentage between 97–100% and e-value range between 1e−120 and 4e−145), *Naegleria* and especially, *Cryptococcus*, which had a higher proportion in the medium anthropogenic pressured wetlands (Fig. S3B).

## Discussion

The wetlands provide multiple socioecological services; however, these have been modified due to the massive deployment of anthropogenic activities. Consequently, it is fundamental to develop strategies to conserve these ecosystems and their functions. Evaluating the current state and frequently monitoring the wetlands are keystones to achieving this objective. One important approach is to assess the organoleptic and physicochemical properties of wetlands as well as classic microbial approaches, and new technologies such as next generation sequencing of markers to determine the microbial composition. All these methods combined might provide a holistic insight of the ecosystem. In this study, a complete evaluation of seven wetlands located at different points in Bogotá, the highly populated capital of Colombia, allowed us to evaluate a first timepoint inherent properties of wetlands associated with different levels of human affectations and the possible correlation between them.

The organoleptic properties, especially the apparent color of the water body and its turbidity, are key factors reflecting anthropogenic activities such as domestic and/or industrial processes, including discharge of wastes which could generate eutrophication or an increase in the productivity of the ecosystem^36^. At the same time, these properties are correlated with the physicochemical characteristics assessed such as the hardness, alkalinity, pH, and others, which also determine the productivity of the ecosystem^37^. Productivity favors the overgrowth of some flora species such as the invasive macrophyte *Eichhornia crassipes*, commonly known as water hyacinth, which has been previously linked to external input of wastes that increases the input of nutrients and overproduction of this plant^38^. This restricts the photosynthesis, leading to deoxygenation shading the water column and thus impacting other aquatic organisms^38^. Additionally, other negative impacts of the overproduction of these macrophytes have been described, such as creating viable conditions for the existence some disease vectors affecting the human communities nearby these altered ecosystems^39^. This invasive species as other flora invasive species easily noticeable during the sample collection, especially in the wetlands classified as highly and moderate pressured wetlands assessed here. This highlights the direct consequences of the human burden in these ecosystems and the impact on the physicochemical and biological properties of these.

The parameters here assessed give us an insight into the ecological dynamic of the differentially pressured wetlands assessed. Specifically, a relationship can be observed between some physicochemical, biological, the presence of some microorganisms and the level of human affectation. One of the highly intervened wetlands, Jaboque, presents a hardness equal to zero, the presence of nitrites (Table 1), a high presence of coliforms (Table 2), and a high abundance of prokaryotic organisms such as *Polynucleobacter* (Fig. 2). These properties potentially differentiate this ecosystem from the others (Figs. 3 and 4). Tthe urban influence, proximity to urban settlements and direct sewage discharge observed during the sample collection, and previously reported by the governmental entities^40^ highlight that the methodology implemented here could represent e a current state of the ecosystem and the impact of external factors. Moreover, as described above, there is a clear differentiation in the prokaryotic and eukaryotic composition between the levels of human burden in the wetlands, which represents a potential methodology to evaluated and monitor the ecosystem status and the possible changes associated with threatening factors such as human direct and indirect impacts.

On the other hand, the assessed oxygen concentration (Table 1) provides insight into the ecological dynamics related to the photosynthesis process and external factors such as rain and decomposition of plants and animals^41^. Wetlands with higher values and thereby higher photosynthetic activity where Chicú and Córdoba (medium anthropogenic pressure), in which the relationship between the amount of dissolved oxygen and the concentration of carbon dioxide reflects the respiration process, decomposition of microorganisms and the chemical oxidation of organic matter^36^, acting as some of the main factors of oxygen consumption in water. Additionally, CO_2_ is an indicator of the ecosystem's metabolism since it is a precursor of photosynthesis. Furthermore, it is important to highlight photosynthetic activity related to Cyanobacteria microorganisms present with high abundance in all the assessed wetlands, being the only prokaryotes capable of carrying out photosynthesis and possibly influencing the values of dissolved oxygen reported for each wetland.

The characteristics described above could partially reflect the status and quality of the ecosystem as adequate or healthy, with higher photosynthetic activity associated with a remarkable presence of plants and photosynthetic microorganisms. On the contrary, higher values of dissolved CO_2_ represent a higher degradation of fauna and flora and with other physicochemical parameters that could represent the unhealthiness and the concern regarding the ecosystem status. However, further studies need to be design and carry out to prove the relationship between this variables, microbial composition, and water/ecosystem quality.

Additional traditional microbiological analysis revealed the presence of coliforms, particularly *E. coli*, in all the wetlands tested using both non-selective and selective agar. The values were outside the criteria established by the national resolution^10^, indicating poor water quality with respect to microbial analysis. This pattern was further supported by high-throughput 16S-rRNA sequencing (Fig. 4A). It can be inferred that coliform values may be influenced by the presence of birds that inhabit or visit wetlands^42^, and/or due to wastewater contamination^41^. This finding indicates that the water from these wetlands is not bacteriologically safe for human consumption^36^. Consequently, there is a need for periodic surveillance and regulatory actions by the governmental entities due to the potential threat to human health that these poor-quality wetlands pose, particularly with the current implementation of these aquatic sources for activities such as agricultural, livestock and recreational services. Furthermore, the molecular approaches used in this study provide an important insight into new efficient and highly informative techniques to implement in national water quality surveillance resolutions.

The amplicon-based sequencing of the 16S-rRNA and 18S-rRNA markers is a potential approach which could provide further information related to the different dynamics and the effect of external factors in the ecosystems, associated to the changes of these microbial communities’ composition and the role that they have in the ecosystem. Our taxonomic identification results of the bacterial composition of the evaluated wetlands partially describe the temporal dynamics of these ecosystems, where the presence of some Betaproteobacteria such as *Malikia* and *Rhodoferax* represent the dominant organisms, as reported previously in water bodies (Fig. 2A)^43,44^, and especially, as representative genera associated with a fresh water clade^45^. Additionally, these genera are associated with some biological functions in the ecosystems, where *Malikia* has been reported as a possible polyhydroxyalkanoate- and polyphosphate-accumulating bacteria^46^, similar to the genus *Pseudomonas*, which has been reported as another polyphosphate-accumulating bacteria^47^. Furthermore, it has been reported that *Malikia* could represent a potential keystone in aquatic ecosystems impacted by wastewater effluent discharges^48^, which explain the possible relationship between the increase in the relative abundance of this genus with the medium pressured wetlands (Fig. 2A). On the other hand, other highly abundant bacterial genera in the high-pressured wetlands are *Candidatus megaira* and *Polynucleobacter* (Fig. 2A), the first one associated to methane-cycling archaea, conducting anaerobic oxidation of the methane using nitrate^49^, and the second one previously classified as an indicator of urban influence in other water bodies^50^. These findings partially describe the dynamics of the evaluated wetlands where the presence of some bacterial communities provide an insight into the current environmental characteristics and the external human contributions to the prevalence/abundance of these microorganisms, where an important presence of nitrates and phosphates explain the abundance of bacteria capable of oxidizing these compounds. Moreover, the higher abundance of the *Polynucleobacter* genus in the high anthropogenic pressured wetland confirms the anthropogenic impact in these ecosystems, as reported in other impacted water bodies in Bogotá, Colombia^18^.

The eukaryotic microbial composition of aquatic ecosystems has been reported briefly and limitedly, primarily focusing on microorganisms related to human health concern. However, there is insufficient information regarding a complete microorganism composition report for aquatic ecosystems or the microbiological status of these ecosystems and the possible effects of external natural and/or human interventions. We described the eukaryotic composition of the wetlands evaluated here, where the most abundant phyla were Ciliophora, Fungi, Mesomycetozoa, Opalozoa and Sagenista (Fig. 2B). Moreover, there were some genera of prokaryotic and eukaryotic organisms such as *Aspergillus*, *Penicillium*, *Escherichia-Shigella*, *Clostridium*, *Aeromonas*, *Cryptococcus*, *Candida*, *Blastocystis*, and *Naegleria* reported in one or more wetlands assessed (Fig. 4). These microorganisms are of particular interest due the pathogenic risk that represent for human and animal health^51–54^, also previously reported in other water bodies^18^. Specifically, the subtype of *Blastocystis* reported here for the Córdoba wetland, ST17, one of the less frequent subtypes reported and associated with cattle^55^, provides further insight into the wetland dynamics in which the possible presence of cattle (commonly observed in the Bogotá wetlands surroundings) and the direct discharge of their feces in the water body could contribute to the presence of this potential pathogenic microorganism. This finding supports the necessity of a frequent monitoring and prevention of these discharges due the potential threat to human and animal health, as mentioned above, emphasizing the necessity for a review, modification and/or design of new regulations.

Moreover, sewage discharge in the wetlands and the subsequent input of nutrients leads to the development of anaerobic environments that favor the presence of some pathogenic bacteria, which could be ingested directly from the wetland sediments or through aquatic invertebrates by birds, causing avian botulism and therefore the death of the affected birds^56^. Some authors have observed correlations between differentially intervened aquatic ecosystems, changes in environmental characteristics, eutrophication, and botulism, enhancing the development of avian pathogens^56,57^ like the organisms found in the wetlands assessed here, *Clostridium spp*., *Escherichia coli, Aeromonas spp.,* and *Pseudomonas spp.* (Figs. 2 and 4), which could affect the avian fauna of the wetlands, where some of them are endemic and represent an important percentage of the diversity that the wetlands hold.

The findings of this study highlight the crucial need of continuous and appropriate surveillance of wetlands, especially those experiencing significant anthropogenic impact. These depositions cause substantial modifications to the characteristics of wetlands, affecting organoleptic and physicochemical parameters, as well as the microbial ecology context. Consequently, the services provided by these ecosystems are impacted, posing potential risks to the health of both humans and animals. Therefore, it is essential for these programs to incorporate approaches that align with the One Health perspective. This is particularly important within the context of international conventions, such as Ramsar, which emphasize the significance of implementing these approaches in key ecosystems based on designated sites. Moreover, it describes a valuable tool for implementing inclusive ecosystem monitoring efforts.

Finally, although this study represents a single point in the time, it provides a starting point for comparison and association with external natural and anthropogenic pressures that could modify these reference parameters and generate potential health risk and loss of the ecosystem services. Furthermore, the amplicon-based sequencing approach has been employed in different characterization efforts of microbial communities in various microbiome studies, making it a potential source of information in the characterization efforts of ecosystems within a holistic surveillance objective. However, the methodology used in this study is a unique and comprehensive proposal for water column quality monitoring that should be further evaluated.

## Conclusions

The present study provides a groundbreaking evaluation of seven Bogotá wetlands, of which five are designated as Ramsar sites within the Complex of Urban Wetlands. These wetlands exhibit varying degrees of human impact. To accomplish this evaluation, a combination of traditional physicochemical and microbiological culture dependent analyses, alongside next-generation sequencing of two molecular markers, were used to characterize the prokaryotic and eukaryotic microbial communities. This comprehensive approach allows for the most current and thorough understanding of these aquatic ecosystems. The results underscore the effectiveness of this approach in assessing the condition of these environments, which offer a wide range of ecological services, including biological regulation, as well as recreational and well-being benefits. Our findings describe the current status and microbial composition associated with the level of human intervention of these ecosystems, providing an approach to monitoring the level of water quality threat to public health. Notably, the Jaboque, Juan Amarillo and Santa Ma. Del Lago wetlands lacked optimal physicochemical parameters for a biological equilibrium and had high abundance of microorganisms associated with fecal contamination and human pathogens, highlighting the importance of these wetlands. However, the identification of pathogens was shared in other wetlands emphasizing the need for continuous monitoring of these water bodies that are surrounded by areas where rural residents are moving into the city and settle around these wetlands.

### Supplementary Information

Supplementary Legends.

Supplementary Figure S1.

Supplementary Figure S2.

Supplementary Figure S3.

Supplementary Information 1.

Supplementary Information 2.

Supplementary Information 3.

Supplementary Information 4.

## Data Availability

The datasets generated and analyzed during the current study are available in the ENA repository under the project number PRJEB57705.
