# Supplementary material for: Characterization of microbial communities in seven wetlands with different anthropogenic burden using Next Generation Sequencing in Bogotá, Colombia
Source: Sci Rep. 2023 Oct 9;13:16973. doi: 10.1038/s41598-023-42970-w (PMC10562456; doi:10.1038/s41598-023-42970-w)
Supplement: Supplementary file 1 — Supplementary Legends. [file 41598_2023_42970_MOESM1_ESM.docx]

**Fig. S1.** Rarefaction curves for the different wetland. A. For the procaryotic community (16S-rRNA). B. For the eucaryotic community (18S-rRNA).

**Fig. S2**. Relative abundance of all procaryotic communities analyzed in this study. This information was obtained by amplicon-based sequencing of 16S-rRNA.

**Fig. S3.** Abundance representation of some microorganisms of interest due their potential impact inhuman and animal health, A. Prokaryotic organisms, and B. Eukaryotic organisms.
