# Supplementary figures and images for: Characterization of microbial communities in seven wetlands with different anthropogenic burden using Next Generation Sequencing in Bogotá, Colombia
Source: Sci Rep. 2023 Oct 9;13:16973. doi: 10.1038/s41598-023-42970-w (PMC10562456; doi:10.1038/s41598-023-42970-w)

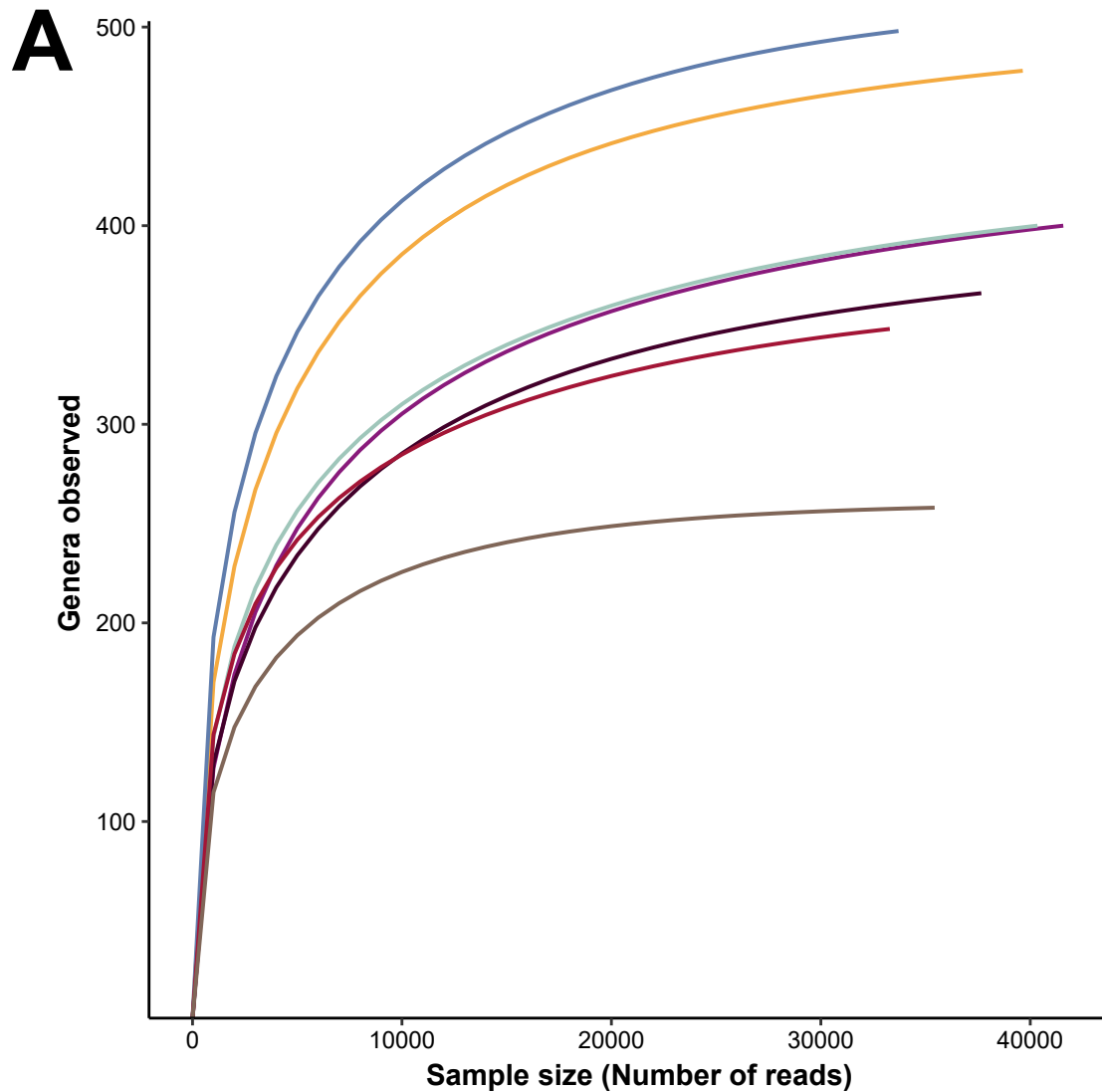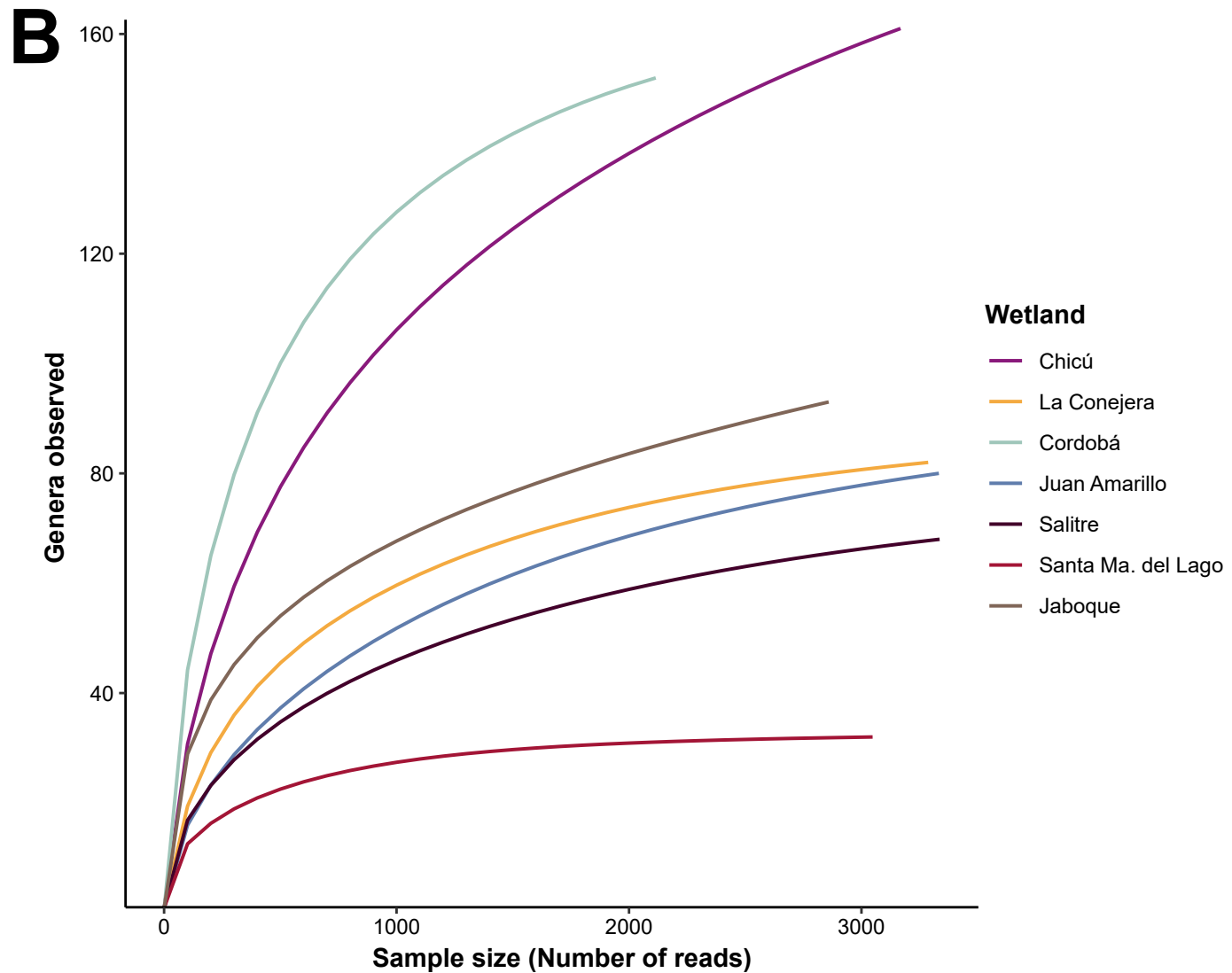

Supplement: Supplementary file 2 — Supplementary Figure S1. [file 41598_2023_42970_MOESM2_ESM.pdf]

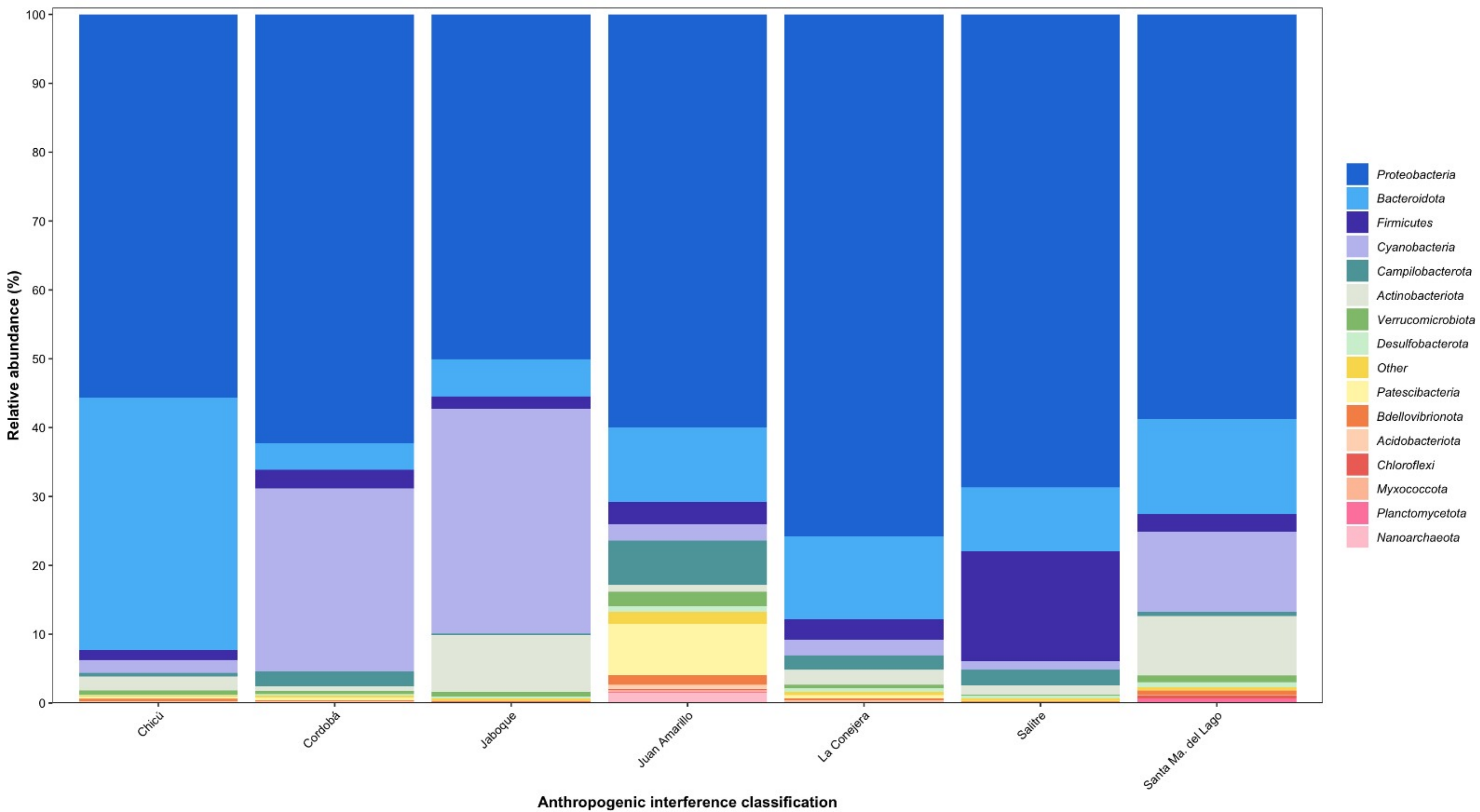

Supplement: Supplementary file 3 — Supplementary Figure S2. [file 41598_2023_42970_MOESM3_ESM.pdf]

**A**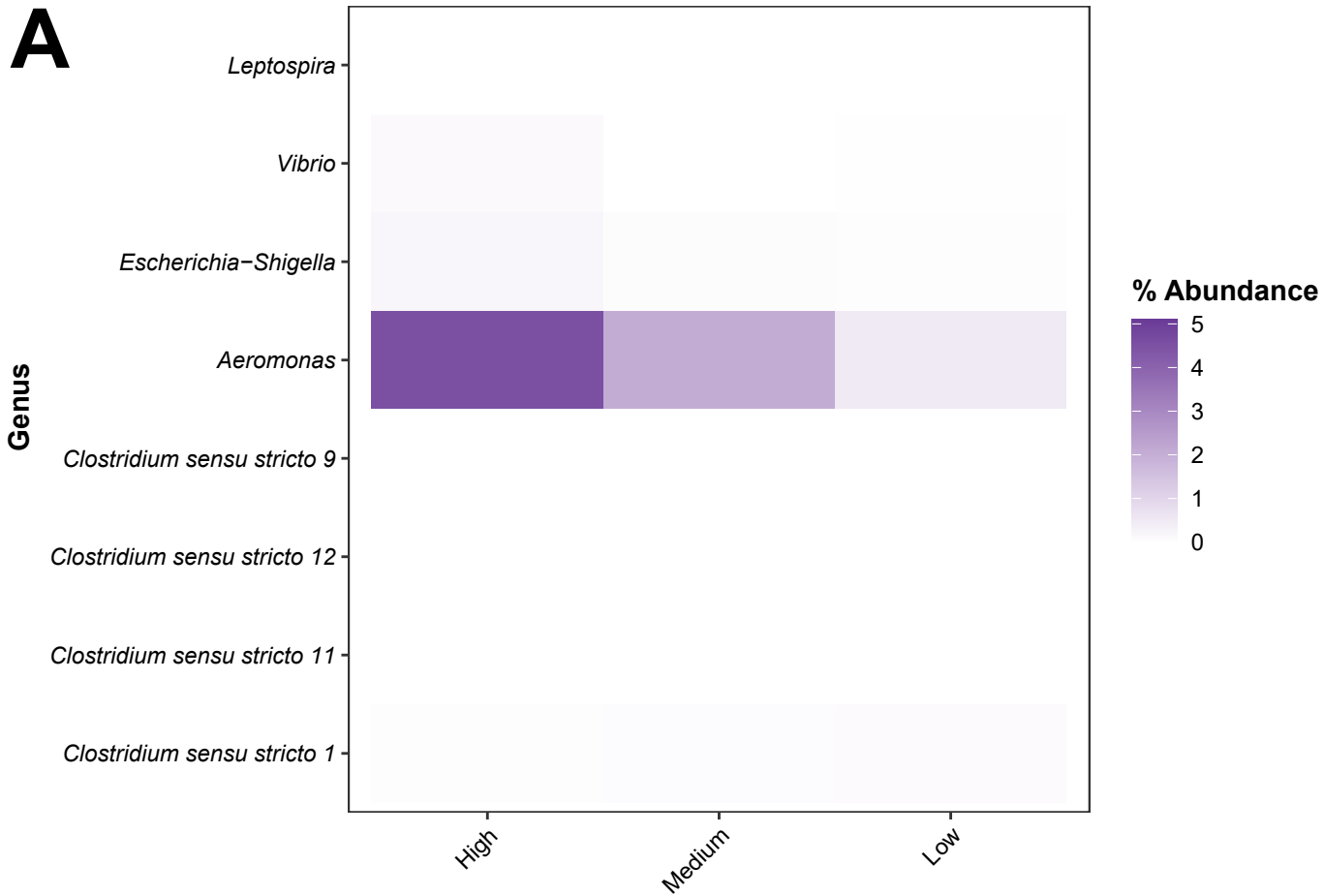**B**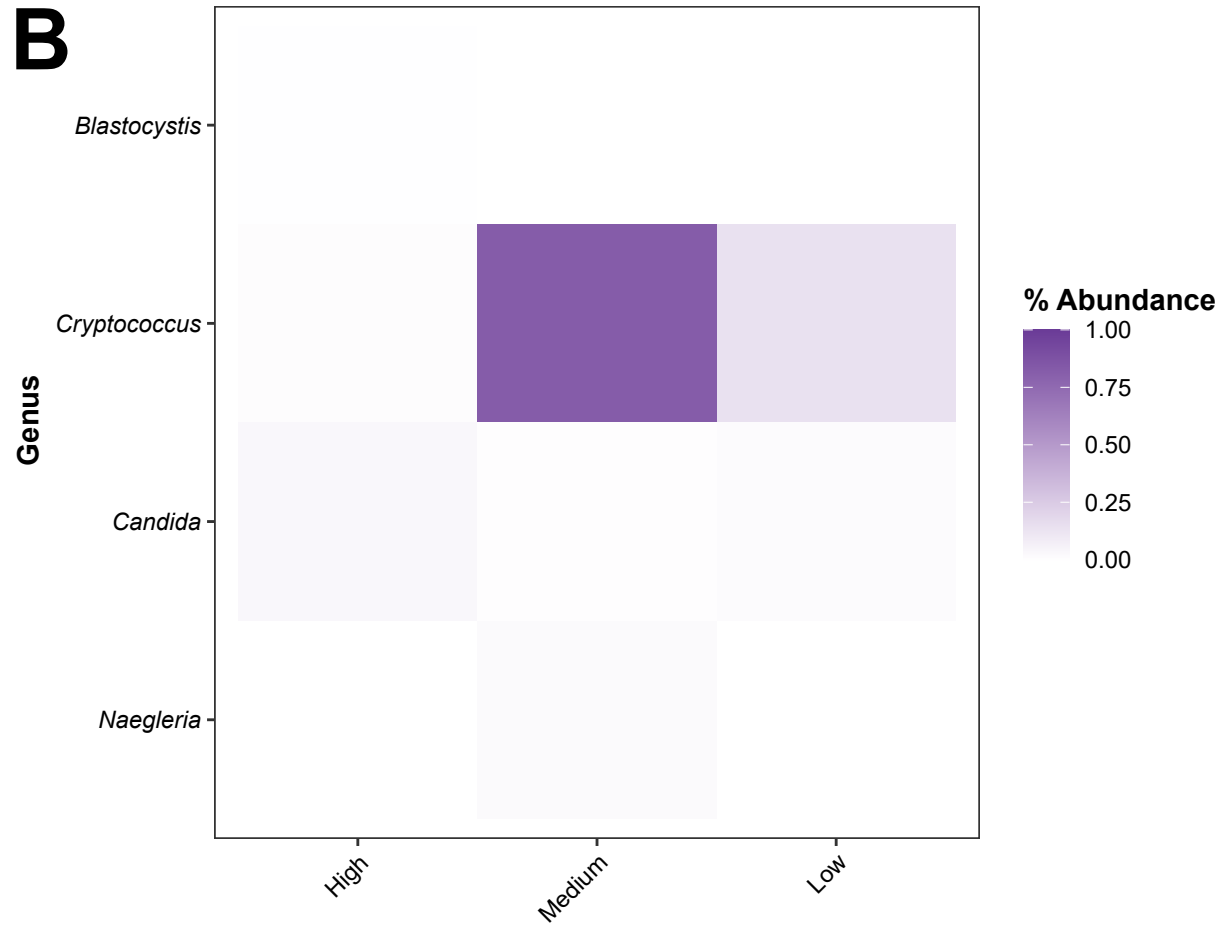

Supplement: Supplementary file 4 — Supplementary Figure S3. [file 41598_2023_42970_MOESM4_ESM.pdf]
