# Supplementary material for: Characterization of microbial communities in seven wetlands with different anthropogenic burden using Next Generation Sequencing in Bogotá, Colombia
Source: Sci Rep. 2023 Oct 9;13:16973. doi: 10.1038/s41598-023-42970-w (PMC10562456; doi:10.1038/s41598-023-42970-w)
Supplement: Supplementary file 6 — Supplementary Information 2. [file 41598_2023_42970_MOESM6_ESM.pdf]

CAR 06/11/2020 11:07
Al Contestar cite este No.: **20202180858**
Origen: Dirección de Recursos Naturales
Destino:OMAIRA BERNATE GIL
Anexos: Fol: 1

Bogotá,

Doctora
OMAIRA BERNATE GIL
Profesional de investigación
Carrera 26 # 63B - 48 Sede Quinta de Mutis
omaira.bernate@urosario.edu.co
Bogotá

ASUNTO: Radicado 20201166396: Consulta permisos recolección de muestras.

Respetada Doctora

Por medio de la presente me permito informarle que, conforme a la normatividad ambiental vigente, no se requiere algún tipo de permiso ambiental para la toma de muestras en los humedales de Bogotá y Cundinamarca. No obstante, para la realización de esta actividad es importante coordinar con las autoridades municipales para solicitar el acceso a estas estructuras ecológicas las cuales por lo general se localizan en predios de propiedad de los municipios y en espacio publico.

Cordialmente,

RICHARD GIOVANNY VILLAMIL MALAVER
Director de Recursos Naturales

Respuesta a: 20201166396 del 22/10/2020

Elaboró: Rodolfo Cujaban Salinas / DRN

Firmado digitalmente y estampado cronológicamente por CERTICAMARA S.A. Artículo 28 de la Ley 527 de 1999 y Artítulo 5 de la Resolución CAR 3783 de 2018.
